# Hexokinase 2 expression in apical enterocytes correlates with inflammation severity in patients with inflammatory bowel disease

**DOI:** 10.1186/s12916-024-03710-7

**Published:** 2024-10-23

**Authors:** Saskia Weber-Stiehl, Jan Taubenheim, Lea Järke, Christoph Röcken, Stefan Schreiber, Konrad Aden, Christoph Kaleta, Philip Rosenstiel, Felix Sommer

**Affiliations:** 1 Institute of Clinical Molecular Biology, University of Kiel, Rosalind-Franklin-Straße 12, Kiel, 24105 Germany; 2 Institute of Experimental Medicine, University of Kiel & University Hospital Schleswig-Holstein, Michaelisstr. 5, Kiel, 24105 Germany; 3 Department of Pathology, University Hospital Schleswig-Holstein, Campus Kiel, Arnold-Heller-Straße 3/House U33, Kiel, 24105 Germany; 4 Department of Internal Medicine I, University Hospital Schleswig-Holstein, Campus Kiel, Kiel, 24105 Germany

**Keywords:** Inflammation, Hexokinase, HK2, Human biopsies

## Abstract

**Background:**

Inflammation is characterized by a metabolic switch promoting glycolysis and lactate production. Hexokinases (HK) catalyze the first reaction of glycolysis and inhibition of epithelial HK2 protected from colitis in mice. HK2 expression has been described as elevated in patients with intestinal inflammation; however, there is conflicting data from few cohorts especially with severely inflamed individuals; thus, systematic studies linking disease activity with HK2 levels are needed.

**Methods:**

We examined the relationship between HK2 expression and inflammation severity using bulk transcriptome data derived from the mucosa of thoroughly phenotyped inflammatory bowel disease (IBD) patients of two independent cohorts including both subtypes Crohn’s disease (CD) and ulcerative colitis (UC). Publicly available single-cell RNA sequencing data were analyzed, and immunofluorescence staining on colonic biopsies of unrelated patients with intestinal inflammation was performed to confirm the RNA-based findings on cellular and protein level.

**Results:**

HK2 expression gradually increased from mild to intermediate inflammation, yet strongly declined at high inflammation scores. Expression of epithelial marker genes also declined at high inflammation scores, whereas that of candidate immune marker genes increased, indicating a cellular remodeling of the mucosa during inflammation with an infiltration of HK2-negative immune cells and a loss of terminal differentiated epithelial cells in the apical epithelium—the main site of HK2 expression. Normalizing for the enterocyte loss clearly identified epithelial HK2 expression as gradually increasing with disease activity and remaining elevated at high inflammation scores. HK2 protein expression was mostly restricted to brush border enterocytes, and these cells along with HK2 levels vanished with increasing disease severity.

**Conclusions:**

Our findings clearly define dysregulated epithelial HK2 expression as an indicator of disease activity in intestinal inflammation and suggest targeted HK2-inhibition as a potential therapeutic avenue.

**Supplementary Information:**

The online version contains supplementary material available at 10.1186/s12916-024-03710-7.

## Background

Prevalence of inflammatory bowel disease (IBD) is rising globally causing severe health issues and drastically reducing quality of life. These diseases are multifactorial with complex interactions of multiple genetic and environmental factors [1]. Despite decades of intense research, the exact etiology of IBD remains mostly unknown limiting treatment options [2]. In recent years, it became evident that ongoing inflammation is characterized by changes in metabolic activity, in particular glucose metabolism [3–5]. Hexokinases (HK) catalyze the first and irreversible step of glycolysis—the phosphorylation of glucose to glucose-6-phosphate—and are therefore crucial for glucose metabolism and maintenance of homeostatic body functions. In mammals, five HK isoenzymes have been identified: HK1, HK2, HK3, GCK (glucokinase), and HKDC1 (HK domain containing 1). Each of these HK isoenzymes displays specific tissue expression patterns and glucose affinities. HK2 is of particular interest as it is the most abundant HK member within the intestine, exerts high HK activity, responds to various internal as well as external stimuli, and shows elevated levels during inflammation [6]. Recently, we further revealed an upregulated expression of HK2 specifically in inflamed compared to non-inflamed tissue of the same patient, irrespective of the type of intestinal inflammation, and demonstrated that ablation of HK2 in intestinal epithelial cells (IEC) protects from acute intestinal inflammation, suppresses cell death, and alters mitochondrial function [6]. These findings placed HK2 as a molecular target to treat intestinal inflammation. However, when expanding our research about the role of HK2 in intestinal inflammation, we encountered transcriptome datasets with contradictory findings of either an unaltered HK2 expression or even downregulation, especially in severely inflamed patients undergoing bowel resection [7]. We therefore aimed to investigate the cause of this apparent contradiction with the goal to clarify the relation between HK2 expression and intestinal inflammation. Here, we show that with increasing inflammation severity the intestinal mucosa is gradually remodeled, which comprises a partial loss of the apical epithelium—the primary source of HK2 expression—and a simultaneous infiltration of immune cells. Normalizing for this cellular remodeling clearly demonstrates a gradual upregulation of HK2 expression with severity of intestinal inflammation.

## Methods

### Human studies

We used sigmoid colon mucosal transcriptomic data and fixed intestinal specimen of two independent large longitudinal clinical studies, namely the EMED [8] (191 samples) and FUTURE [9] (87 samples) cohorts (trial IDs: EudraCT number 2016–000205-36 and ClinicalTrials.gov NCT02694588), for which accompanying disease activity scores (Harvey-Bradshaw Index (HBI)/Mayo Score) were available (see Additional file 1: Table S1 for cohort clinical characteristics). Note that multiple samples were collected from each patient. Sample origin was incorporated in all following analyses. The HBI [10] is used to quantify disease activity in patients suffering from Crohn’s disease (CD), one of the two IBD subtypes. Here, variables such as general well-being, abdominal pain and abdominal mass with each having scores of 0–3, as well as the number of liquid stools per day (1 point per stool), and other complications (1 point per complication) yield the “open-ended” total score (Additional file 2: Table S2). The Mayo score is used for ulcerative colitis (UC), the other clinical IBD subtype. This scoring system accounts for general well-being, rectal bleeding, endoscopic results, and stool frequency with scores of 0–3 per category for an overall score ranging from 0 to 12 (Additional file 2: Table S2). To facilitate a direct comparison of both scores despite them being “open-ended” and “discrete,” we calculated a general inflammation score by setting the highest score in the dataset for each HBI and Mayo score to 1 and scaled the score of each patient accordingly (see Additional file 2: Table S2).

### Transcriptome data analysis

RNA sequencing data derived from mucosal biopsies of patients with various grades of intestinal inflammation were retrieved from two previously published IBD cohorts [8, 9]. Read counts were transformed to transcripts per million (TPM) values to normalize for differential sequencing depths among samples. TPM data were then plotted per sample against the inflammation score using R (version 4.2.2) and ggplot2 (3.4.3). Trendlines were calculated including all data points and using the distance to the actual location as a weight to enable a robust calculation and avoiding overfitting especially in areas of scarce sampling. To test for an association of gene expression to changes in the inflammation score, we used variance-stabilization transformation of raw read counts and fitted linear mixed models with the following form: vst(geneExpression) ~ Age + Sex + Diagnosis + InflammationScore. Statistical analyses were performed using R (version 4.2.2) [11] with the following packages: lm4 (version 1.1–31) [12], lmerTest (version 3.1–3) [13], car (version 3.0–13) [14], ggplot2 (version 3.3.6) [15], and DESeq2 (version 1.38.3) [16].

### Cellular deconvolution

We deconvoluted the bulk RNA data using the MuSiC package (version 1.0.0) [17] with default values. As reference, we used a single-cell dataset from UC patients described in a study by Smillie et. al. [18]. The original single-cell dataset was split in “epithelial,” “immunogenic,” and “stromal” subsets, which we rejoined in our analysis. Due to limited resolution in deconvolution approaches and to reduce cell diversity, we pooled ontogenetically closely related cell types (Additional file 3: Table S3). We restricted the analysis to the eight most abundant cell types.

### Single-cell RNA sequencing

Single-cell data was obtained from the Single Cell Portal (accession SCP259) initially described by Smillie and colleagues [18]. The different cell populations were rejoined and general quality controls were performed. In short, we filtered cells with read counts below 1000 (low quality). Furthermore, we removed cells with less than 200 or with more than 2720 (twofold of the standard deviation of expressed genes across all cells) differently expressed genes to account for spurious sequencing depth and removal of duplicates. All cells with a mitochondrial RNA content above 5% were also removed. For plotting, gene expression values where log-transformed. Plotting and quality filtering was performed in R (version 4.2.2) using ggplot2 (version 3.4.3) and Seurat (version 4.4.0) [19].

### Immunofluorescence

Five-micrometer sections of paraffin-embedded intestinal biopsies were deparaffinized with Xylol substitute (Roth), incubated in citrate buffer for 3 min, and subsequently blocked in 5% BSA-PBS and 0.2% TritonX for 30 min. Primary anti-E-cadherin (1:400 in 1% BSA, #3195, Cell Signaling Technology) and anti-Hexokinase 2 (1:500 in 1% BSA, Cat# NBP2-02272, Novus Biologicals, Colorado, US) antibodies were incubated overnight. Sections were washed, incubated with secondary antibodies (Alexa Fluor 488 goat anti mouse, Invitrogen, A32731 and Alexa Fluor 555 goat anti rabbit, Invitrogen, A21430) for 45 min at room temperature. DAPI (1:40,000 in PBS, D9542, Sigma Aldrich, St. Louis, US) was used for DNA counterstaining. Slides were mounted using antifade mounting media (DAKO, Hovedstaden, Denmark). The quantitative analysis was performed using fluorescence microscopy for stained samples using the imager Z1 microscope (ZEISS, Jena, Germany) and ZEN software (version 3.0). Images were taken by a digital camera system (AxioCam HrC/HrM, Zeiss, Jena, Germany) and ApoTome (ZEISS, Jena, Germany). Fluorescence signal intensity was measured using the Fiji/ImageJ software.

### Statistics

General statistical analyses were performed using the GraphPad Prism 9 (GraphPad Software Inc., La Jolla, USA). For pairwise comparisons, the Mann–Whitney *U* test was used, whereas for multiple comparisons, one-way ANOVA with false discovery rate (FDR) correction were performed. Data are shown as mean ± standard error of the mean (SEM). A *p*-value of ≤ 0.05 was considered as significant (*). A *p*-value of ≤ 0.01 was considered as strongly significant (**) and *p*-value of ≤ 0.001 as highly significant (***). For longitudinal data, linear mixed effect models were fitted where gene expression was used as dependent variable, while inflammation score and patient ID were fixed and random factors. The models were fitted using lme4 (version 1.1–31), and statistical testing for coefficients was performed using lmerTest (version 3.1–3). Model validity was tested against a null-model using log-likelihood ratio tests, and model assumption was evaluated by diagnostic plotting of model residuals, data point influence strength, and random factor fitting.

## Results

### Epithelial HK2 expression increases with high inflammation scores

**Fig. 1:**
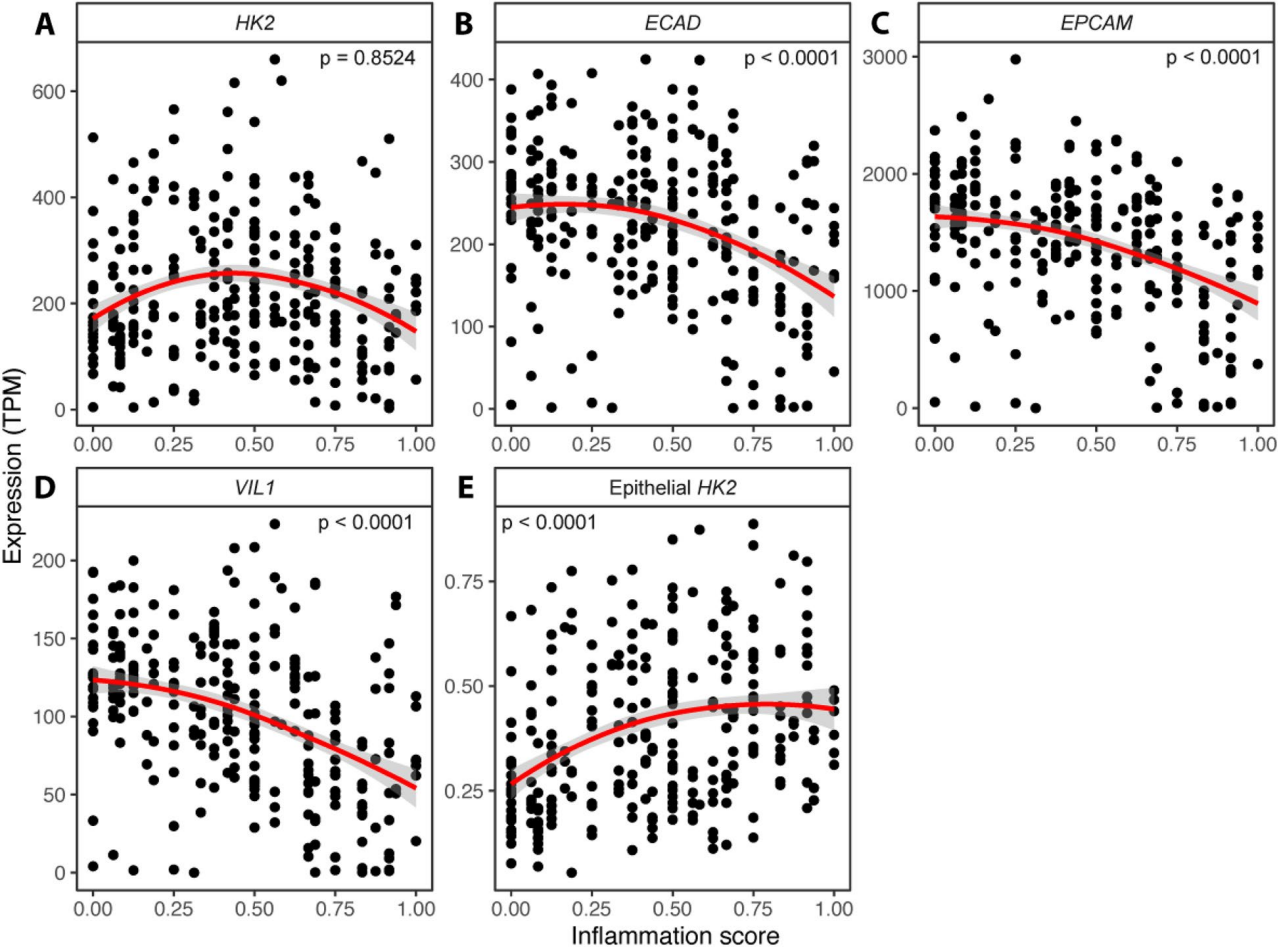
Epithelial HK2 expression correlates with inflammation severity. **A**–**D** Expression (TPM, transcript per million) of *HK2* (**A**) and the epithelial marker genes *ECAD* (**B**), *EPCAM* (**C**), and *VIL1* (**D**) in the sigmoid colon mucosa of patients with various degrees of gut inflammation. Note that at high inflammation scores (> 0.4) expression of *HK2* and the epithelial marker genes all decrease indicating epithelial erosion. The inflammation score was calculated as a scaled Harvey-Bradshaw index (Crohn’s disease) or Mayo score (ulcerative colitis) to accommodate both disease types. **E** *HK2* expression increases with inflammation scores after normalization to epithelial marker gene expression. The red lines represent the mean expression trendline with the grey area indicating its 95% confidence interval. The number in the upper left/right corner represents the *p* value for the correlation between gene expression and inflammation score as determined by linear mixed model

To determine a possible correlation of HK2 expression and disease activity, we analyzed RNA sequencing (RNA-seq) data derived from sigmoid colon biopsies of patients suffering from IBD of two independent clinical studies [8, 9], for which accompanying disease activity scores for each sample were available (Additional file 1: Table S1 and Additional file 2: Table S2). This analysis revealed that HK2 expression initially increased gradually reaching peak expression at mid disease activity (inflammation score ~ 0.4) and then declined with high inflammation scores (Fig. 1A). As within the intestine HK2 is mainly expressed by epithelial cells of the apical mucosa (Additional file 4: Fig. S1), we hypothesized that with very high levels of inflammation these apical epithelial cells and therefore also the main site of HK2 expression might be lost due to shedding and apoptosis or transdifferentiation resulting in decreased HK2 expression levels in bulk RNA-seq data. To investigate this hypothesis, we assessed the expression levels of three different epithelial marker genes, namely E-cadherin (*ECAD*), epithelial cell adhesion molecule (*EPCAM*), and Villin1 (*VIL1*). Supporting our hypothesis, the expression levels of these epithelial markers were all declining with increasing inflammation severity (Fig. 1B–D), which indicates progressive epithelial damage caused by the inflammation. To account for this epithelial loss, we then normalized the expression of HK2 to that of these epithelial markers (mean of the individual TPM values of *ECAD*, *EPCAM*, and *VIL1*), thereby yielding an epithelial HK2 expression. Importantly, epithelial HK2 expression gradually increased in correlation to disease activity until medium inflammation scores and then remained elevated at high inflammation scores (Fig. 1E). To test whether the observed expression changes were unique to the combined clinical cohorts, we also performed these analyses separately for each clinical cohort (Additional file 5: Fig. S2). Importantly, we observed for both clinical cohorts the same changes in *HK2*, *ECAD*, *EPCAM*, *ECAD*, and epithelial HK2 expression. We also tested for potential differences between the two main types of IBD, Crohn’s disease (CD), and ulcerative colitis (UC) and found similar responses both for CD and UC (Additional file 6: Fig. S3). Finally, correlation of *HK2* and epithelial *HK2* expression with disease severity was confirmed using linear regression models (Additional file 7: Fig. S4 and Additional file 8: Table S4).

### Immune cell infiltration during intestinal inflammation

**Fig. 2:**
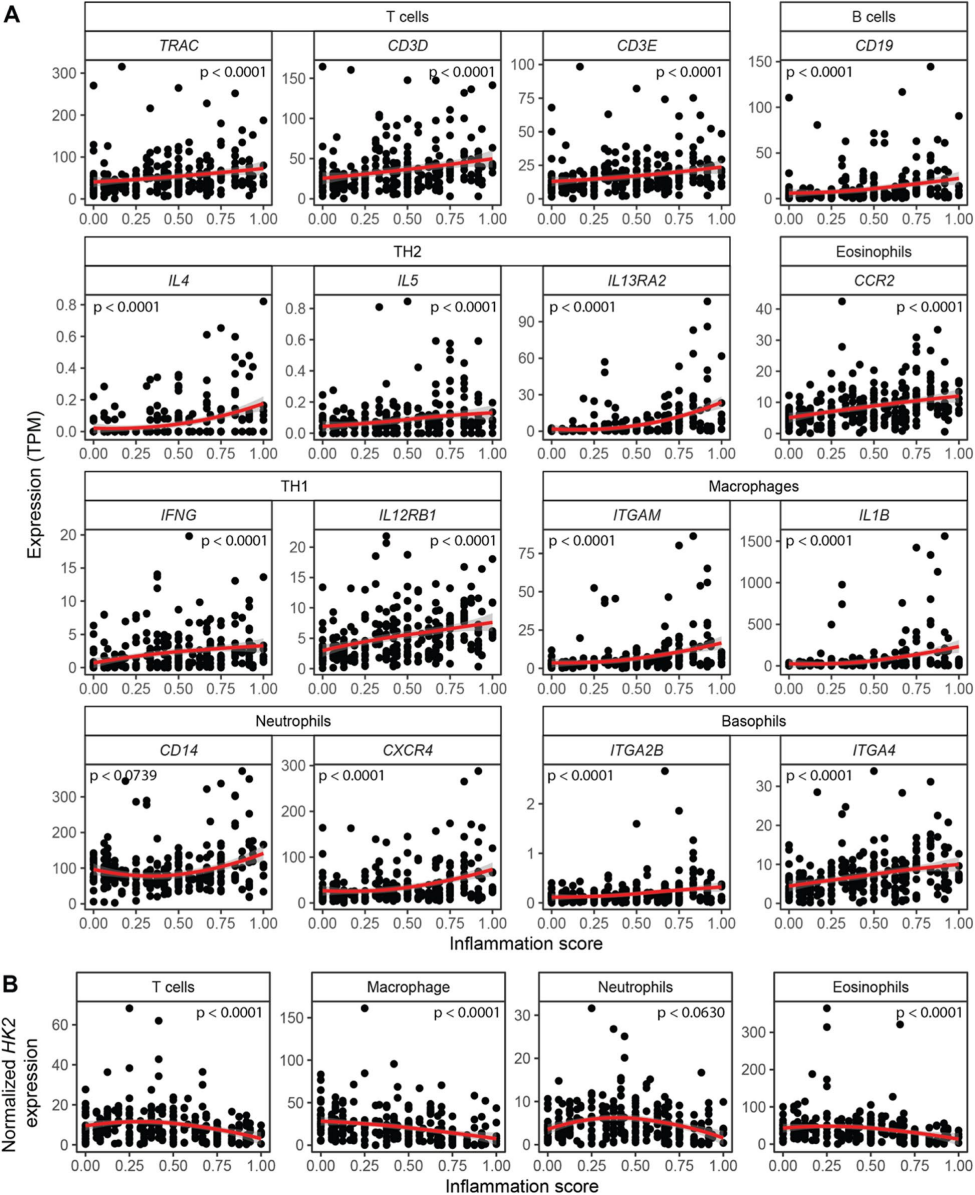
Expression of candidate immune marker genes increases with inflammation severity. Expression of selected marker genes, which are characteristic for individual immune cell types, were evaluated in patient sigmoid colon biopsies. Expression of virtually all candidate immune marker genes increase with inflammation severity indicating an expansion of immune cells—a known feature of intestinal inflammation. The red lines represent the mean expression trendline with the grey area indicating its 95% confidence interval. The number in the upper left/right corner represents the *p* value for the correlation between gene expression and inflammation score as determined by linear mixed model

Another source for cellular remodeling during inflammation is tissue infiltration by immune cells. Using the bulk RNA-seq data we also investigated the proportions of various immune cell types in relation to disease severity. First, we performed a candidate gene-based analysis and chose several immune marker genes to assess infiltration of T cells, T helper 1 (Th1) cells, T helper 2 (Th2) cells, B cells, basophils, neutrophils, eosinophils, and macrophages (Fig. 2A). In contrast to *HK2* and the epithelial marker genes, the expression of these individual immune cell genes increased with the inflammation score. However, slight alterations in the expression patterns could be observed, mostly depending on the immune cell type. Expression of the T cell marker genes *TRAC*, *CD3D* and *CD3E*, the B cell marker gene *CD19*, the eosinophil marker gene *CCR2*, and the neutrophil marker genes *CD14* and *CXCR4* as well as the basophil marker genes *ITGA2B* and *ITGA4* all gradually increased in a linear fashion with the inflammation score (Fig. 2). In contrast, the marker genes for Th2 (*IL4*, *IL5*, *IL13*), Th1 (*IFNG*, *IL12*) cells, and macrophages (*ITGAM*, *IL1B*) all only displayed biphasic expression patterns with a first phase characterized by slight increases in expression until an inflammation score of approximately 0.5 and a second phase at higher disease severity characterized by larger changes in their expression. The greatest expression changes were detected for the macrophage marker genes *ITGAM* and *IL1B* suggesting the greatest relative increase of these cells with increasing disease activity. Normalizing *HK2* expression to the candidate immune cell genes revealed gradual declines with increasing inflammation scores for all tested immune cell types indicating that the immune cell infiltration leads to more cells present in the mucosa, which do not or only express very little *HK2* (Fig. 2B).

**Fig. 3:**
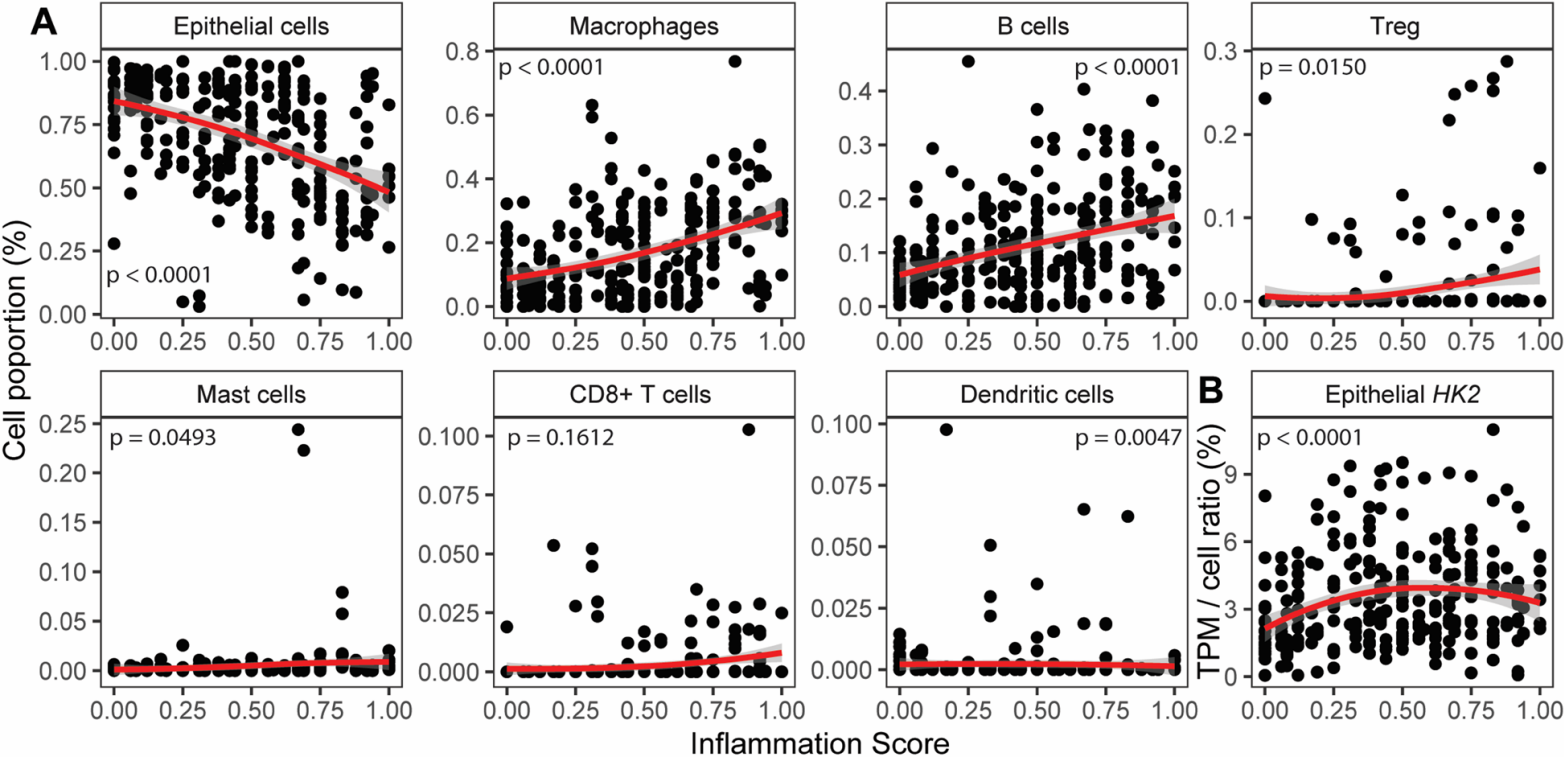
Deconvolution of mucosal transcriptomes demonstrates cellular remodeling during inflammation. **A** MuSiC cell deconvolution package was used to estimate ratios of the most abundant cell types based on the sigmoid colon mucosal patient transcriptomes. Notably, epithelial cell abundance is decreasing with inflammation, whereas abundances of macrophages and B cells, the two most frequent immune cell types that combine for approximately 35% proportion, are increasing with inflammation severity. Therefore, cell deconvolution supports the candidate gene analyses indicating mucosal cellular remodeling characterized by a reduction of epithelial cells and an expansion of immune cells with increasing inflammation severity. **B** *HK2* expression increases with inflammation scores after normalization to epithelial cell abundance. The red lines represent the mean expression trendline with the grey area indicating its 95% confidence interval. The number in the upper left/right corner represents the *p* value for the correlation between gene expression and inflammation score as determined by linear mixed model

Next, we moved from the candidate gene-based to a systematic cellular deconvolution of the bulk RNA-seq data using MuSiC [17]. This program uses a reference single-cell dataset and cell type-specific expression profiles to derive the abundance of the individual cell types from bulk RNA-seq data. We restricted this analysis to the eight most abundant cell types. The proportion of epithelial cells gradually declined with increasing inflammation scores, whereas the proportion of macrophages, B cells, and regulatory T cells increased (Fig. 3A). The proportion of mast, cytotoxic T cells, and dendritic cells remained mostly unchanged. Therefore, this data indicates a massive gradual cellular remodeling of the intestinal mucosa during inflammation, and the findings of the systematic cellular deconvolution supported those of the candidate gene-based analyses. Furthermore, the drastic decrease in the proportion of epithelial cells with increasing inflammation scores demonstrates again that the main source of *HK2* expression is lost during the disease course. This is therefore also reflected by the outcome that after accounting for the abundance of epithelial cells the normalized epithelial *HK2* expression gradually increased with disease severity (Fig. 3B).

### Apical HK2 expression in mature enterocytes correlates with inflammation severity

**Fig. 4:**
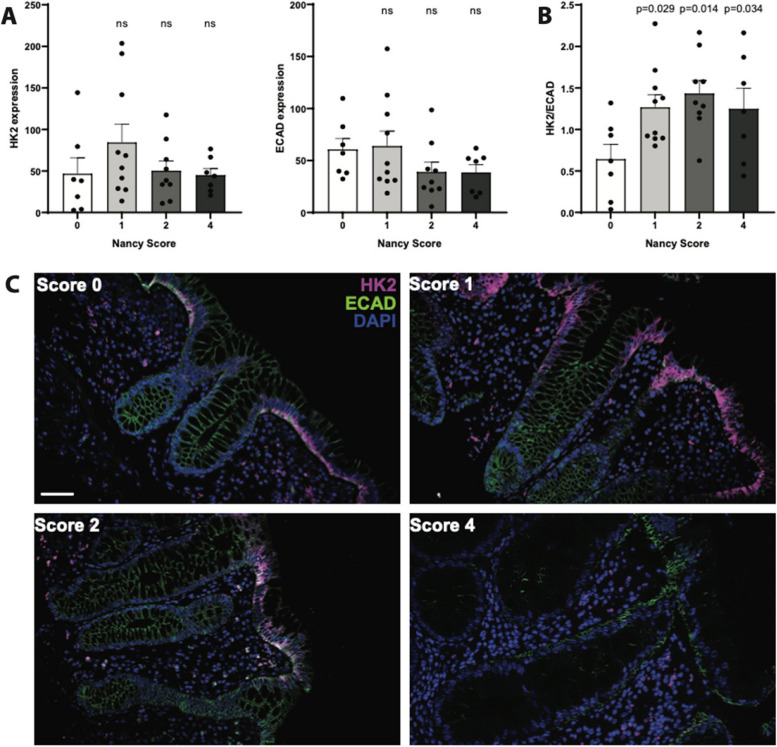
Epithelial HK2 protein expression increases with inflammation severity. Colonic mucosa biopsies of unrelated UC patients were stained for HK2, epithelial cells (ECAD) and nuclei (DAPI). All biopsies were analyzed for classical histological signs of inflammation using the Nancy score (see the “ Methods” section). **A** Quantification of HK2 and ECAD signal intensity in apical intestinal epithelium. Note that HK2 and ECAD level decrease with higher Nancy Scores. **B** HK2 protein expression normalized to ECAD levels. Note that after epithelial normalization HK2 expression increases with inflammation severity, i.e., Nancy score. Significance testing was performed using one-way ANOVA compared to score = 0. **C** Representative images of the multiplex immunofluorescence staining (HK2, ECAD, DAPI). Scale bar indicates 50 μm. *n* = 7–10 per group. Note that microscopic images were taken from areas with rather intact epithelium for a better visualization of the mucosal structure

To validate the transcriptome data and to elucidate the biogeography of the changes in tissue architecture in relation to HK2 expression, we performed immunostainings on colonic biopsies of an independent set of UC patients that had been thoroughly scored for histological disease severity (Nancy score [20]). HK2 protein levels increased non-significantly in mildly inflamed (score 1) compared to non-inflamed specimen (score 0) and remained unaltered at medium or strong inflammation (scores 2 and 4) (Fig. 4A, C). In contrast, protein levels of the epithelial marker E-cadherin (ECAD) were unchanged in mild (score 1) but non-significantly reduced at medium or strong inflammation (scores 2 and 4) compared to non-inflamed control samples (Fig. 4A, C). Notably, structural epithelial damage of inflamed biopsies was detectable with increasing inflammatory scores (Fig. 4C) with loss of epithelial cells and an infiltration of other cells, probably immune cells, into the inflamed mucosa. In addition, many epithelial cells that were not shed but still present at the inflamed site had reduced ECAD expression, which could indicate transdifferentiation of these inflamed apical enterocytes. Normalizing the HK2 protein levels to those of ECAD revealed that epithelial HK2 protein levels (Fig. 4B) significantly increased with inflammation regardless of disease severity (scores 1–4). This epithelial HK2 protein expression in inflamed intestinal mucosal biopsies mirrored and confirmed the transcript patterns of the RNA sequencing analysis.

**Fig. 5:**
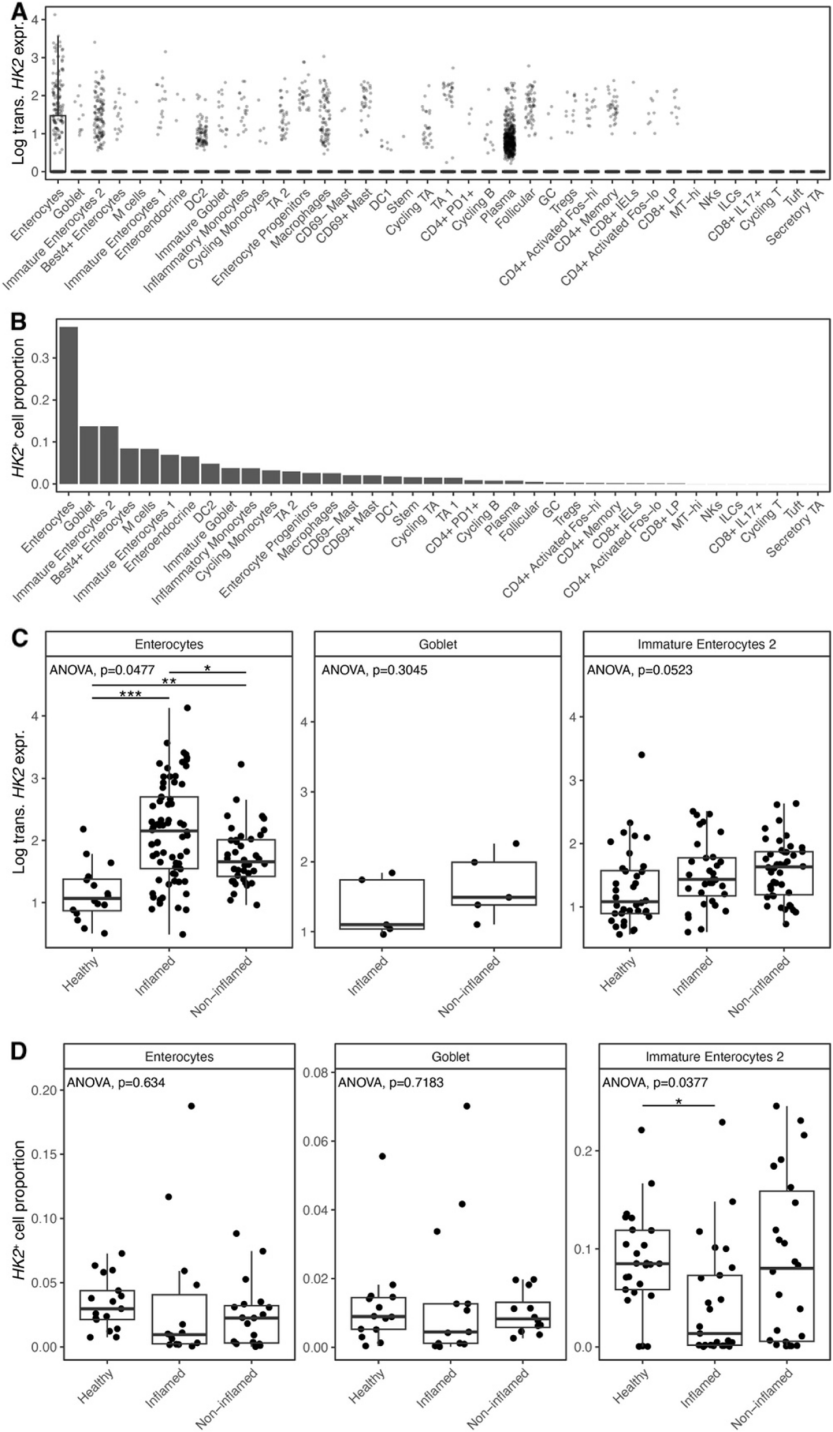
*HK2* is mainly expressed by mature enterocytes and increased during inflammation. *HK2* expression was analyzed in published single-cell RNA sequencing data derived from mucosal biopsies of UC patients [18]. **A** *HK2* expression per cell type. Box plot depicting the median and 25th–75th percentile. Whiskers indicate most extreme points within 1.5-times interquartile range deviance from the median and dots represent samples outside of this interval. Note that only enterocytes express significant *HK2* levels; thus, only here an interval box is visible. **B** Abundances of cell types expressing *HK2*. **C** *HK2* expression in mature and immature enterocytes and goblet cells divided into samples from healthy controls, inflamed and non-inflamed mucosa. **D** Abundances of cell types expressing *HK2* after stratification into disease group. ANOVA *p* values denote whether significant differences among the three groups exist that were then further analyzed by pairwise Wilcoxon tests (*p < 0.05, **p < 0.01, ***p < 0.001)

Furthermore, we used recently published data from single-cell RNA sequencing of mucosal biopsies from IBD patients [18] to investigate the *HK2* expression on a cellular level in relation to intestinal inflammation. This analysis of single-cell RNA sequencing data confirmed our findings from the bulk RNA sequencing and immunofluorescence, as *HK2* was mainly expressed by mature enterocytes and then followed by goblet cells and immature enterocytes (Fig. 5A, B). Next, we checked for *HK2* expression pattern in enterocytes at different inflammatory stages (healthy vs. inflamed vs. non-inflamed) and found that the lowest *HK2* expression can be observed in healthy controls, while cells from inflamed samples showed an increased HK2 expression (Fig. 5C). Non-inflamed samples had intermediate *HK2* expression levels (Fig. 5C), which indicates that HK2 expression is dysregulated in enterocytes of IBD patients also in the absence of an overt inflammation. This pattern of *HK2* expression was also present although less predominant in immature enterocytes and goblet cells. Finally, we looked into the number of these three cell types that are detected in the three health states and found that mature and immature enterocytes are lost during inflammation, while incomplete recovery can be observed in non-inflamed tissue of IBD patients (Fig. 5D). Altogether, the single-cell data therefore supports a cellular reprogramming during inflammation with loss of mature enterocytes (the main cell type of the apical epithelium) and a dysregulated *HK2* expression in inflamed apical epithelial cells.

In summary, both the RNA and protein data clearly demonstrate that epithelial HK2 was overexpressed during intestinal inflammation, which highlights HK2 as an indicator of active disease. Building upon previous findings from murine models that inhibition of HK2 expression either genetically or via the microbial metabolite butyrate protect from experimental colitis [6], these new data now show that indeed HK2 expression is dysregulated in the mucosa of patients with active inflammation and therefore suggest that targeting HK2 may represent a promising approach to suppress intestinal inflammation in humans.

## Discussion

### Epithelial HK2 is a marker for intestinal inflammation

Prevalence of intestinal inflammation is increasing and, thus, there is a growing need for valid disease biomarkers and molecular targets. Despite significant efforts, the cause of chronic intestinal inflammation remains unknown. In a recent study, HK2 was identified as upregulated under inflammatory conditions in mice and humans irrespective of disease subtype (CD, UC, or non-IBD colitis) and suppressing HK2 expression even protected from experimental colitis [6] placing HK2 as a potential disease marker. However, we found other transcriptome datasets generated from severely inflamed patients in which HK2 expression was unaltered or even reduced [7]. Here, we therefore thoroughly investigated the relationship between HK2 expression and disease severity by analyzing transcriptome data derived from intestinal biopsies of patients with intestinal inflammation for which corresponding data on disease severity (HBI, Mayo score, Nancy score) were available. In addition, we performed cellular deconvolution analyses of the RNA sequencing data to infer the changes in cell proportions during inflammation. Finally, we used immunofluorescence staining to clarify the changes in HK2 protein biogeography in the mucosa during inflammation. Using these approaches, we demonstrated that raw HK2 RNA and protein expression at first gradually increased with the inflammation scores, yet after reaching a critical inflammation score, HK2 expression declined again at very severe inflammation. These findings integrate the seemingly conflicting data from previous studies [7, 21–23] by linking HK2 expression to disease severity.

### Cellular remodeling of the intestinal mucosal during inflammation drives overall HK2 level

**Fig. 6:**
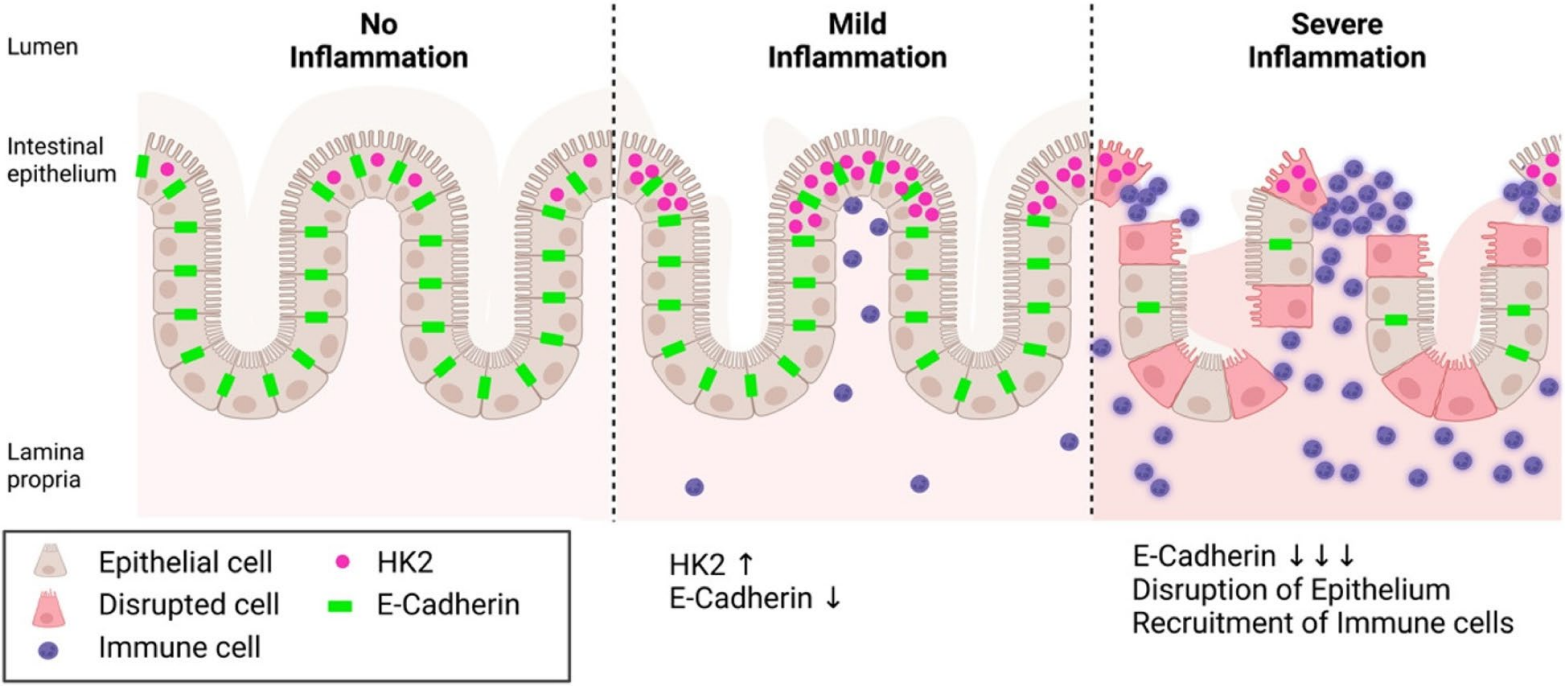
Model of HK2 expression changes in relation to intestinal inflammation severity. HK2 is predominantly expressed by apical epithelial cells and HK2 expression in these cells increases with disease severity. However, during the course of inflammation, the cellular composition of the mucosa changes. In severely inflamed tissue, the epithelium is disrupted, brush border epithelial cells with high levels of HK2 are shed and lost, whereas immune cells with little to no HK2 expression are recruited. Therefore, overall HK2 expression is reduced in bulk tissue samples under severe inflammation despite local high expression in remaining HK2-positive epithelial cells. Created with BioRender.com

We further were able to demonstrate that loss of HK2 expression at high inflammation levels was dependent on the disruption of brush border enterocytes as both the expression of epithelial marker genes were decreasing with the inflammation score and immunofluorescence analyses clearly pointed to a destruction of the apical epithelium—the main site of HK2 expression [24, 25]. These findings are in line with clinical practice, in which epithelial damage is a key criterion to classify an increasing inflammatory state during IBD [26], for example with epithelial erosion being a feature of histological inflammation in the Nancy score. By analyzing single-cell RNA sequencing data derived from an independent set of UC patients, we confirmed that in the gut epithelial cells are the main source of *HK2* expression, both in terms of per cell expression as well as the number of cells contributing to overall expression (Fig. 5). These cells are consequently lost due to increasing inflammation in the single-cell dataset, which would explain the decrease of *HK2* expression at high levels of inflammation [18]. Finally, we were able to deconstruct the simultaneous epithelial erosion and immune cell infiltration into the submucosa during intestinal inflammation from the RNA sequencing data. Based on the overall abundance and their induction (fold change) during inflammation, infiltrating macrophages seemed most relevant, but also other cell types such as B cells increased in proportion. Especially the expansion of macrophages could be important and contributing to the loss of epithelial cells and therefore HK2 expression during inflammation as IL-1β interferes with the tight junction complexes between IECs [27–29] and thereby increases intestinal permeability [30]. Similar to epithelial erosion, immune cell infiltration is a classical feature of inflammation and used in clinical practice to histologically evaluate disease severity, in particular as a feature of the Nancy score [20]. In addition, transdifferentiation of HK2-positive mature enterocytes into other cell types such as HK2-negative immature enterocytes and stem cells [31, 32] or maybe even into mesenchymal cells as during epithelial–mesenchymal transition [33] could also contribute to a reduction of HK2 levels in the inflamed mucosa. In summary, our findings imply that during the course of inflammation, changes in the cellular composition of the mucosa affect the overall bulk HK2 expression. In particular, HK2-positive brush border epithelial cells are lost, whereas HK2-negative immune cells are recruited to the site of inflammation. This cellular restructuring will lead to an overall reduction of HK2 levels, although some remaining brush border epithelial with HK2 expression remain, but their numbers get fewer and fewer (Fig. 6). Overall, we want to highlight the importance of taking into consideration the biogeography and changes in cell type expression ratios, when trying to identify disease biomarkers. This is especially important regarding complex diseases such as intestinal inflammation involving various internal and external factors. Most previous studies used bulk expression data in the search for disease biomarkers or therapeutic targets and therefore may have missed other locally restricted but disease relevant genes like *HK2*. In our analyses, HK2 expression was only significantly associated with disease severity after considering epithelial cell abundance (Fig. 1E and Fig. 3B), and we speculate that this pattern will also hold true for other genes with an apical or otherwise restricted expression. For example, other hexokinases (HK1, HKDC1), proteins responsible for the uptake of dietary nutrients (e.g., SGLT1, SLC15A1) or those contributing to the intestinal epithelial barrier (e.g., CLDN15, ZO-1), also show a predominant or even restricted apical expression and therefore possibly have not yet been identified as disease biomarker during severe intestinal inflammation due to the loss of their primary expression site. Notably, epithelial remodeling not only includes the partial loss of apical enterocytes and infiltration of lymphocytes but also includes hyper-regeneration, metaplasia, and a loss of goblet cells. The advent of single-cell technologies such as single-cell RNA sequencing promise to pave the way for more refined analyses that will enable the discovery of more suitable disease markers and to enhance our understanding of the molecular and cellular mechanisms during disease progression.

### Mitochondrial dysfunction seems to precede histological inflammation

Upon accounting for cellular remodeling during inflammation, epithelial HK2 expression not only correlated with disease severity (Fig. 1E), but also the increase in *HK2* expression even preceded signs of histological inflammation (Fig. 4). These findings therefore support a concept that metabolic reprogramming, in particular a switch from oxidative phosphorylation (OXPHOS) to favoring anaerobic glycolysis linked to mitochondrial dysfunction, controls the transition from homeostasis into an inflammatory response [34]. IBD has been proposed as being a state of energy deficiency [3], which is congruent with metabolizing glucose via anaerobic glycolysis rather than via OXPHOS as the former yields a vastly smaller amount of ATP (4 mol ATP versus 36 mol ATP per mol glucose) potentially leading to an energetically restricted state during chronic inflammation. In recent years, mitochondrial dysfunction in intestinal epithelial cells had been linked to stem cell function, mucosal regeneration, and Paneth cell dysfunction [35–37] along with inflammatory protection potentially by inert immune responses [6]. An important role of mitochondria for inflammation has also been highlighted by genome-wide association studies with IBD patients that identified > 240 genetic variants contributing to disease establishment and pathologies, of which approximately 5% have direct roles in regulating mitochondrial homeostasis [38]. Those mitochondrial IBD risk alleles include a regulator of PPIF (component of the mitochondrial permeability transition pore) [39], SLC22A5 (involved in mitochondrial β oxidation) [40], or ATG16L1 [41] and NOD2 [42], both of which are involved in mitophagy and the removal of dysfunctional mitochondria. Importantly, in a cohort of CD patients, molecular marks of mitochondrial dysfunction could be detected in non-inflamed tissue margins and predicted disease recurrence [18]. This data together with several prior reports [35, 36, 43, 44] support our findings on the regulation of HK2 during inflammation, thus arguing that disturbed epithelial metabolic and mitochondrial function precede tissue inflammation and could potentially even be causal in IBD pathogenesis.

### Targeted HK2 inhibition as potential treatment avenue for intestinal inflammation

Our findings highlight dysregulated HK2 expression, i.e., increased levels, during intestinal inflammation, thus placing a rational for inhibition of HK2 as a novel approach to treat chronic inflammation in IBD patients. However, HK2 carries out important physiological functions in virtually all cells and organs of the body, mainly its metabolic activity in glycolysis but also has a role in pathogen recognition by macrophages [45, 46]. Mice with a ubiquitous deletion of HK2 in the entire body are not viable [47–49], whereas *Hk2*^∆IEC^ mice with a conditional deletion of HK2 only in intestinal epithelial cells do not display any physiological abnormalities [6]. Therefore, inhibition of HK2 during intestinal inflammation should target only the intestine and no other organs. Ideally, inhibition should even only target those cells of the intestine with a dysregulated HK2, namely inflamed terminal differentiated epithelial cells in the apical epithelium with the aim to avoid them starting their apoptotic program and elimination/shedding. The therapeutic value of inhibiting epithelial HK2 had already been demonstrated using the *Hk2*^∆IEC^ mice, which were protected from experimental acute colitis [6]. This study also demonstrated that colonic supplementation of the microbial metabolite butyrate ameliorated colitis in HK2-proficient littermate wildtype mice by suppressing HK2 expression. In humans, however, oral and rectal administration of butyrate initially yielded some promising results with disease ameliorations, but ultimately these clinical trials failed due to side-effects presumably caused by the unphysiologically high local butyrate concentrations [50–52]. These data therefore argue that other approaches are needed to enable a more region-specific inhibition of HK2, for example using capsules that are designed to allow a controlled release of physiological butyrate amounts only in the colon [53]. Alternatively, highly specific HK2 inhibitors such as Benitrobenrazide [54] or “Compd 27” [55] have been developed recently that combined with the aforementioned encapsulation currently seems to be the most specific option of targeted colonic HK2 inhibition. Clinical trials are required to test feasibility and performance of these approaches. Furthermore, temporal and spatial metabolomic analysis of mucosal biopsies from patients with known disease severity could aid in the clarification whether butyrate levels contribute to controlling HK2 expression and inflammation in humans.

### Conclusions

HK2 has not yet been described as IBD risk gene, most probably due to its site-restricted expression, but here we were able to show an association of HK2 and epithelial status during intestinal inflammation, in particular cellular composition driven by infiltration of HK2-negative immune cells and erosion of terminal differentiated epithelial cells in the apical epithelium. Therefore, epithelial HK2 expression correlates with disease severity making it a useful indicator of intestinal inflammation and highlighting the therapeutic potential of targeting HK2. However, ultimately, clinical studies are required to indeed demonstrate the feasibility and efficacy of a HK2-targeted intervention in IBD patients.

## Supplementary Information

Additional file 1: Table S1. Cohort clinical characteristics. Note that the inflammation score represents an integrated metric of the Harvey-Bradshaw Index (HBI) and the Mayo Score, which are used to quantify disease activity in CD and UC, respectively (see the “Mmethods” section and Additional file 2: Table S2 for details on the individual scores and their integration into the inflammation score).

Additional file 2: Table S2. Translation of the HBI and Mayo Score into a general Inflammation Score. The definitions of the HBI and Mayo scores are listed. The Inflammation Score was calculated by scaling each the HBI and Mayo Score from 0-1 and then merging the scores.

Additional file 3: Table S3. Original and pooled deconvolution cell types. Originally classified cell types that are ontogenetically related, e.g., Paneth cells, enterocytes, goblet cells and stem cells, were pooled and classified into “epithelial cells” to reduce cell type diversity.

Additional file 4: Fig. S1. HK2 expression in the intestinal mucosa of mice. Biopsies from small and large intestine were immuno-stained to localize the HK2 protein in the mucosa. Note that HK2 expression is mainly confined to epithelial cells of the apical mucosa both in the small and large intestine. Murine specimens were used to facilitate comparison to human colonic immunostainings (Fig. 4), which is important to contextualize data from in vivo inflammation models, and to enable the best tissue protection and architecture for spatial analyses that rarely is achieved with human intestinal biopsies. Scale bars represent 50 μm.

Additional file 5: Fig. S2. Epithelial HK2 expression correlates with disease severity in both clinical cohorts. Expression data (TPM, transcript per million) of HK2 and the epithelial marker genes ECAD, VIL1 and EPCAM in the intestinal mucosa of patients with various degrees of gut inflammation split per clinical cohort FUTURE and EMED. The red lines 19 represent the mean expression trendline with the grey area indicating its 95% confidence interval.

Additional file 6: Fig. S3: Epithelial HK2 expression correlates with inflammation severity regardless of disease subtype. Expression data (TPM, transcript per million) of HK2 and the epithelial marker genes ECAD, VIL1 and EPCAM in the intestinal mucosa of patients with various degrees of gut inflammation split per disease subtype CD and UC. The red lines represent the mean expression trendline with the grey area indicating its 95% confidence interval.

Additional file 7: Fig. S4. Correlation analysis for HK2 expression and inflammation score. Three different linear mixed effect models (PatientID as random factor) were fitted to the (A) HK2 and the (B) epithelial HK2 expression. First, all points were used to estimate the overall effect of the data and inflammation score. Afterwards the data set was split into “low scores” (inflammation score < 0.5), "high scores” (inflammation score >0.5) and fitted models for these data subsets. While the first model over all data showed no significant association between HK2 expression and inflammation, there is a positive association at “low scores” and a negative association at “high scores” (see also Additional file 8: Table S4) indicating that HK2 expression increases with inflammation during lower disease scores and then decreases at higher disease scores. For epithelial HK2 expression there is a positive association for all data and “low inflammation scores,”, while the at “high inflammation scores” there is no significant association, but epithelial HK2 expression remained elevated. This therefore might indicate a saturation of the epithelial HK2 expression at high inflammation.

Additional file 8: Table S4. Comparison of linear mixed model statistics for the coefficients explaining HK2 and epithelial HK2 expression with inflammation scores for models created with all data, low (score <0.5) or high (score >0.05) inflammation scores only.

## Data Availability

The bulk RNA sequencing data is publicly available at NCBI Gene Expression Omnibus (https://www.ncbi.nlm.nih.gov/geo) under the accession numbers GSE191328 (EMED) and GSE171770 (FUTURE). The single cell RNA sequencing data is publicly available at the Broad Institute Single Cell Portal (http://singlecell.broadinstitute.org) under the accession number SCP259.

## Abbreviations

ANOVA: Analysis of variance
ATG16L1: Autophagy related 16 like 1
ATP: Adenosine triphosphate
BSA: Bovine serum albumin
CCR2: C-C chemokine receptor type 2
CD: Cluster of differentiation
CD: Crohn’s disease
CLDN15: Claudin 15
CXCR4: C-X-C chemokine receptor type 4
DAI: Disease activity index
DAPI: 4′,6-Diamidino-2-phenylindole
DNA: Deoxyribonucleic acid
DSS: Dextran sodium sulfate
ECAD: E-cadherin
EPCAM: Epithelial cell adhesion molecule
FDR: False discovery rate
GCK: Glucokinase
GO: Gene ontology
H&E: Hematoxylin and eosin
HBI: Harvey-Bradshaw index
HK: Hexokinase
HK2: Hexokinase 2
HKDC1: HK domain containing 1
IBD: Inflammatory bowel disease
IEC: Intestinal epithelial cell
IL: Interleukin
ITGA2B: Integrin alpha 2b
ITGAM: Integrin alpha M
NOD2: Nucleotide-binding oligomerization domain-containing protein 2
OXPHOS: Oxidative phosphorylation
PBS: Phosphate-buffered saline
PPIF: Peptidyl-prolyl cis–trans isomerase
SEM: Standard error of the mean
SGLT1: SLC15A1
SLC22A5: Solute carrier family 22 member 5
TBS: Tris-buffered saline
Th: T helper
TNF: Tumor necrosis factor
TPM: Transcripts per million
TRAC: T cell receptor alpha constant
TUNEL: Terminal deoxynucleotidyl transferase dUTP nick end la-beling
UC: Ulcerative colitis
VIL1: Villin1
WT: Wild-type
ZO-1: Zonula occludens-1
