# Supplementary material for: Hexokinase 2 expression in apical enterocytes correlates with inflammation severity in patients with inflammatory bowel disease
Source: BMC Med. 2024 Oct 23;22:490. doi: 10.1186/s12916-024-03710-7 (PMC11515617; doi:10.1186/s12916-024-03710-7)
Supplement: Supplementary file 1 — Additional file 1: Table S1. Cohort clinical characteristics. Note that the inflammation score represents an integrated metric of the Harvey-Bradshaw Index (HBI) and the Mayo Score, which are used to quantify disease activity in CD and UC, respectively (see the “Mmethods” section and Additional file 2: Table S2 for details on the individual scores and their integration into the inflammation score). [file 12916_2024_3710_MOESM1_ESM.docx]

**Additional file 1: Table S1: Cohort clinical characteristics.** Note that the inflammation score represents an integrated metric of the Harvey-Bradshaw Index (HBI) and the Mayo Score, which are used to quantify disease activity in CD and UC, respectively (see method section and Additional file 2: Table S2 for details on the individual scores and their integration into the inflammation score).

| **Cohort** | **EMED** | **FUTURE** | **Combined** | **CD** | **UC** |
| --- | --- | --- | --- | --- | --- |
| Patients | 41 | 16 | 57 | 28 | 29 |
| Samples | 191 | 87 | 278 | 141 | 137 |
| Age in years, min - max (median) | 21 - 65 (26) | 16 -74 (36) | 16 - 74 (35) | 16 - 74 (35) | 18 - 65 (35) |
| Sex assigned at birth | 57 female  30 male | 119 female  72 male | 176 female  102 male | 95 female  56 male | 81 female  56 male |
| Smoking | 5 smoker  17 previous  65 non-smoker | 53 smoker  30 previous  108 non-smoker | 58 smoker  47 previous  173 non-smoker | 40 smoker  25 previous  76 non-smoker | 18 smoker  22 previous  97 non-smoker |
| Age at first diagnosis in years, min - max (median) | 6 - 60 (22) | 3 -61 (25) | 3 - 61 (24) | 3 - 61 (22) | 13 - 60 (26) |
| Time since first diagnosis in years, min - max (median) | 0 - 20 (4) | 0 - 29 (9) | 0 - 29 (6) | 0 - 27 (8) | 0 - 29 (5) |
| Inflammation score, min - max (median) | 0 - 1 (0.375) | 0 - 1 (0.625) | 0 - 1 (0.4375) | 0 - 1 (0.5) | 0 - 1 (0.375) |
