# Supplementary material for: Hexokinase 2 expression in apical enterocytes correlates with inflammation severity in patients with inflammatory bowel disease
Source: BMC Med. 2024 Oct 23;22:490. doi: 10.1186/s12916-024-03710-7 (PMC11515617; doi:10.1186/s12916-024-03710-7)
Supplement: Supplementary file 2 — Additional file 2: Table S2. Translation of the HBI and Mayo Score into a general Inflammation Score. The definitions of the HBI and Mayo scores are listed. The Inflammation Score was calculated by scaling each the HBI and Mayo Score from 0-1 and then merging the scores. [file 12916_2024_3710_MOESM2_ESM.docx]

**Additional file 2: Table S2: Translation of the HBI and Mayo Score into a general Inflammation Score.** The definitions of the HBI and Mayo scores are listed. The Inflammation Score was calculated by scaling each the HBI and Mayo Score from 0-1 and then merging the scores.

| **HBI** |  |  |
| --- | --- | --- |
|  |  |  |
| **Criterium** | **Score** | |
| General well-being | Very well | 0 |
|  | Slightly poor | 1 |
|  | Poor | 2 |
|  | Very poor | 3 |
|  | Terrible | 4 |
| Abdominal pain | None | 0 |
|  | Mild | 1 |
|  | Moderate | 2 |
|  | Severe | 3 |
| Abdominal mass | None | 0 |
|  | Dubious | 1 |
|  | Definite | 2 |
|  | Definite with tenderness | 3 |
| Stool frequency | Number of liquid stools | 1 for each lquid stool per day |
| Complications | Arthralgia, Uveitis, Erythema nodosum, Apthous ulcers, Pyoderma gangrenosum, Anal fissure, New fistula, Abscess | 1 point each |

| **Mayo Score** |  |  |
| --- | --- | --- |
|  |  |  |
| **Criterium** | **Score** | |
| Number of stools per day | Normal for the patient | 0 |
|  | 1-2 stools more than normal | 1 |
|  | 3-4 stools more than normal | 2 |
|  | 5+ stools more than normal | 3 |
| Rectal blood | No blood | 0 |
|  | Blood streaks in less the half of evacuations | 1 |
|  | Evidence of fresh blood in most of the evacuations | 2 |
|  | Bowel movements with fresh blood | 3 |
| Endoscopy | Normal or inactive disease | 0 |
|  | Mild disease (erythema, decreased vascular pattern, mild friability) | 1 |
|  | Moderate disease (marked erythema, lack of vascular pattern, friability, erosions) | 2 |
|  | Severe disease (spontaneous bleeding, ulceration) | 3 |
| Global medical asessment | Normal | 0 |
|  | Mild disease | 1 |
|  | Moderate disease | 2 |
|  | Severe disease | 3 |

| **Calculation of inflammation score** | | |  |  |  |  |  |  |  |  |
| --- | --- | --- | --- | --- | --- | --- | --- | --- | --- | --- |
|  |  |  |  |  |  |  |  |  |  |  |
|  |  |  |  |  |  |  |  |  |  |  |
| **HBI** | **InfScore** |  | **Mayo** | **InfScore** |  | **>>>** |  | **InfScore** | **HBI** | **Mayo** |
| 0 | 0.00 |  | 0 | 0.00 |  | merged |  | 0.00 | 0 | 0 |
| 1 | 0.06 |  | 1 | 0.08 |  |  |  | 0.06 | 1 |  |
| 2 | 0.12 |  | 2 | 0.17 |  |  |  | 0.08 |  | 1 |
| 3 | 0.18 |  | 3 | 0.25 |  |  |  | 0.12 | 2 |  |
| 4 | 0.24 |  | 4 | 0.33 |  |  |  | 0.17 |  | 2 |
| 5 | 0.29 |  | 5 | 0.42 |  |  |  | 0.18 | 3 |  |
| 6 | 0.35 |  | 6 | 0.50 |  |  |  | 0.24 | 4 |  |
| 7 | 0.41 |  | 7 | 0.58 |  |  |  | 0.25 |  | 3 |
| 8 | 0.47 |  | 8 | 0.67 |  |  |  | 0.29 | 5 |  |
| 9 | 0.53 |  | 9 | 0.75 |  |  |  | 0.33 |  | 4 |
| 10 | 0.59 |  | 10 | 0.83 |  |  |  | 0.35 | 6 |  |
| 11 | 0.65 |  | 11 | 0.92 |  |  |  | 0.41 | 7 |  |
| 12 | 0.71 |  | 12 | 1.00 |  |  |  | 0.42 |  | 5 |
| 13 | 0.76 |  |  |  |  |  |  | 0.47 | 8 |  |
| 14 | 0.82 |  |  |  |  |  |  | 0.50 |  | 6 |
| 15 | 0.88 |  |  |  |  |  |  | 0.53 | 9 |  |
| 16 | 0.94 |  |  |  |  |  |  | 0.58 |  | 7 |
| 17 | 1.00 |  |  |  |  |  |  | 0.59 | 10 |  |
|  |  |  |  |  |  |  |  | 0.65 | 11 |  |
|  |  |  |  |  |  |  |  | 0.67 |  | 8 |
|  |  |  |  |  |  |  |  | 0.71 | 12 |  |
|  |  |  |  |  |  |  |  | 0.75 |  | 9 |
|  |  |  |  |  |  |  |  | 0.76 | 13 |  |
|  |  |  |  |  |  |  |  | 0.82 | 14 |  |
|  |  |  |  |  |  |  |  | 0.83 |  | 10 |
|  |  |  |  |  |  |  |  | 0.88 | 15 |  |
|  |  |  |  |  |  |  |  | 0.92 |  | 11 |
|  |  |  |  |  |  |  |  | 0.94 | 16 |  |
|  |  |  |  |  |  |  |  | 1.00 | 17 | 12 |
