# Supplementary material for: Hexokinase 2 expression in apical enterocytes correlates with inflammation severity in patients with inflammatory bowel disease
Source: BMC Med. 2024 Oct 23;22:490. doi: 10.1186/s12916-024-03710-7 (PMC11515617; doi:10.1186/s12916-024-03710-7)
Supplement: Supplementary file 3 — Additional file 3: Table S3. Original and pooled deconvolution cell types. Originally classified cell types that are ontogenetically related, e.g., Paneth cells, enterocytes, goblet cells and stem cells, were pooled and classified into “epithelial cells” to reduce cell type diversity. [file 12916_2024_3710_MOESM3_ESM.docx]

**Additional file 3: Table S3: Original and pooled deconvolution cell types.** Originally classified cell types that are ontogenetically related, e.g. Paneth cells, enterocytes, goblet cells and stem cells, were pooled and classified into “epithelial cells” to reduce cell type diversity.

| **Original cell type description** | **Grouped into cell type** |
| --- | --- |
| TA 1 | epis |
| TA 2 | epis |
| Enterocyte Progenitors | epis |
| Immature Goblet | epis |
| Immature Enterocytes 1 | epis |
| Immature Enterocytes 2 | epis |
| Cycling TA | epis |
