# Supplementary material for: Hexokinase 2 expression in apical enterocytes correlates with inflammation severity in patients with inflammatory bowel disease
Source: BMC Med. 2024 Oct 23;22:490. doi: 10.1186/s12916-024-03710-7 (PMC11515617; doi:10.1186/s12916-024-03710-7)
Supplement: Supplementary file 4 — Additional file 4: Fig. S1. HK2 expression in the intestinal mucosa of mice. Biopsies from small and large intestine were immuno-stained to localize the HK2 protein in the mucosa. Note that HK2 expression is mainly confined to epithelial cells of the apical mucosa both in the small and large intestine. Murine specimens were used to facilitate comparison to human colonic immunostainings (Fig. 4), which is important to contextualize data from in vivo inflammation models, and to enable the best tissue protection and architecture for spatial analyses that rarely is achieved with human intestinal biopsies. Scale bars represent 50 μm. [file 12916_2024_3710_MOESM4_ESM.docx]

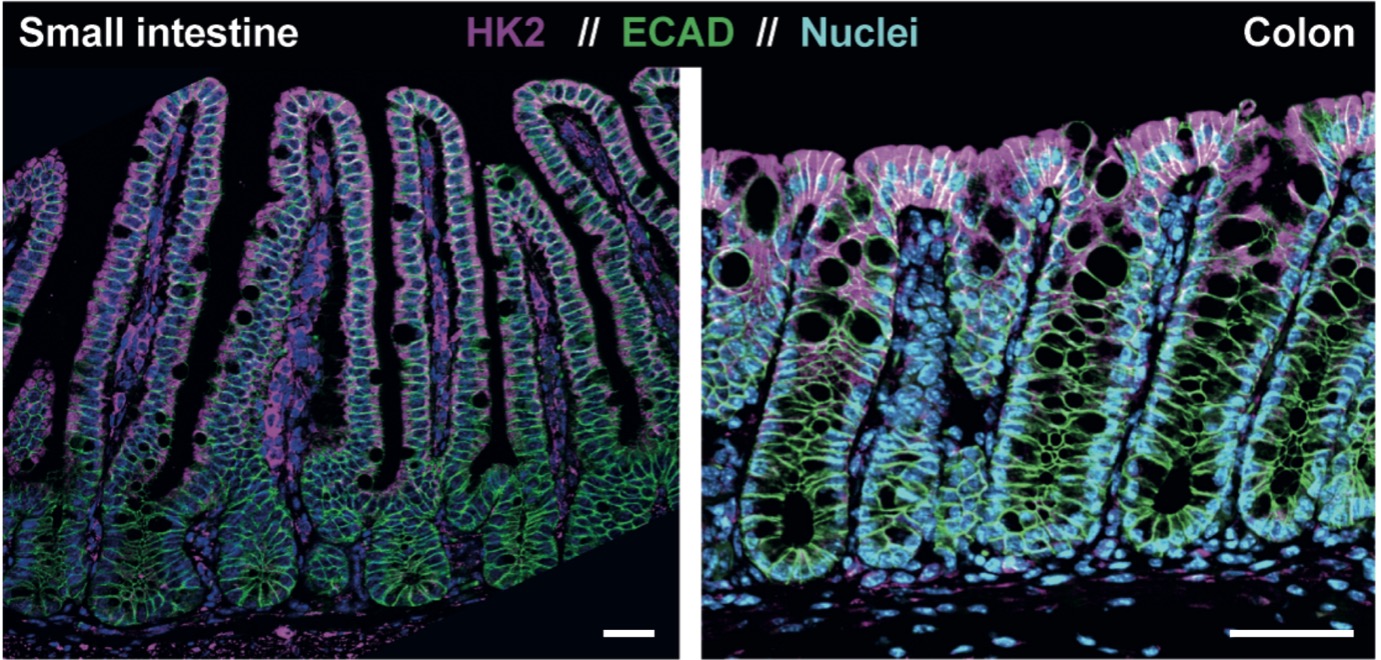


**Additional file 4: Figure S1: HK2 expression in the intestinal mucosa of mice.** Biopsies from small and large intestine were immuno-stained to localize the HK2 protein in the mucosa. Note that HK2 expression is mainly confined to epithelial cells of the apical mucosa both in the small and large intestine. Murine specimens were used to facilitate comparison to human colonic immunostainings (Figure 4), which is important to contextualize data from *in vivo* inflammation models, and to enable the best tissue protection and architecture for spatial analyses that rarely is achieved with human intestinal biopsies. Scale bars represent 50 µm.
