# Supplementary material for: Hexokinase 2 expression in apical enterocytes correlates with inflammation severity in patients with inflammatory bowel disease
Source: BMC Med. 2024 Oct 23;22:490. doi: 10.1186/s12916-024-03710-7 (PMC11515617; doi:10.1186/s12916-024-03710-7)
Supplement: Supplementary file 6 — Additional file 6: Fig. S3: Epithelial HK2 expression correlates with inflammation severity regardless of disease subtype. Expression data (TPM, transcript per million) of HK2 and the epithelial marker genes ECAD, VIL1 and EPCAM in the intestinal mucosa of patients with various degrees of gut inflammation split per disease subtype CD and UC. The red lines represent the mean expression trendline with the grey area indicating its 95% confidence interval. [file 12916_2024_3710_MOESM6_ESM.docx]

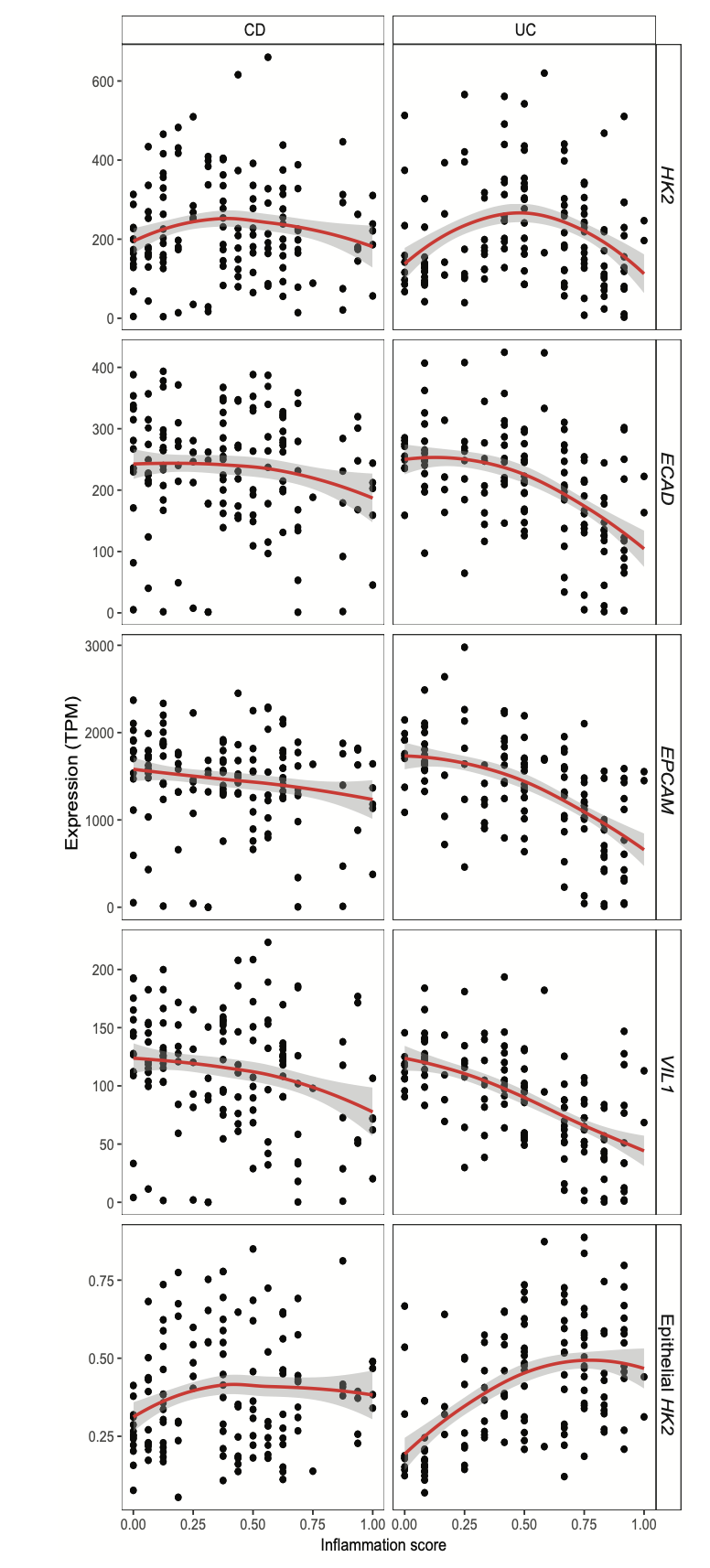


**Additional file 6: Figure S3: Epithelial *HK2* expression correlates with inflammation severity regardless of disease subtype.** Expression data (TPM, transcript per million) of *HK2* and the epithelial marker genes *ECAD, VIL1* and *EPCAM* in the intestinal mucosa of patients with various degrees of gut inflammation split per disease subtype CD and UC. The red lines represent the mean expression trendline with the grey area indicating its 95% confidence interval
