# Supplementary material for: Hexokinase 2 expression in apical enterocytes correlates with inflammation severity in patients with inflammatory bowel disease
Source: BMC Med. 2024 Oct 23;22:490. doi: 10.1186/s12916-024-03710-7 (PMC11515617; doi:10.1186/s12916-024-03710-7)
Supplement: Supplementary file 7 — Additional file 7: Fig. S4. Correlation analysis for HK2 expression and inflammation score. Three different linear mixed effect models (PatientID as random factor) were fitted to the (A) HK2 and the (B) epithelial HK2 expression. First, all points were used to estimate the overall effect of the data and inflammation score. Afterwards the data set was split into “low scores” (inflammation score < 0.5), "high scores” (inflammation score >0.5) and fitted models for these data subsets. While the first model over all data showed no significant association between HK2 expression and inflammation, there is a positive association at “low scores” and a negative association at “high scores” (see also Additional file 8: Table S4) indicating that HK2 expression increases with inflammation during lower disease scores and then decreases at higher disease scores. For epithelial HK2 expression there is a positive association for all data and “low inflammation scores,”, while the at “high inflammation scores” there is no significant association, but epithelial HK2 expression remained elevated. This therefore might indicate a saturation of the epithelial HK2 expression at high inflammation. [file 12916_2024_3710_MOESM7_ESM.docx]

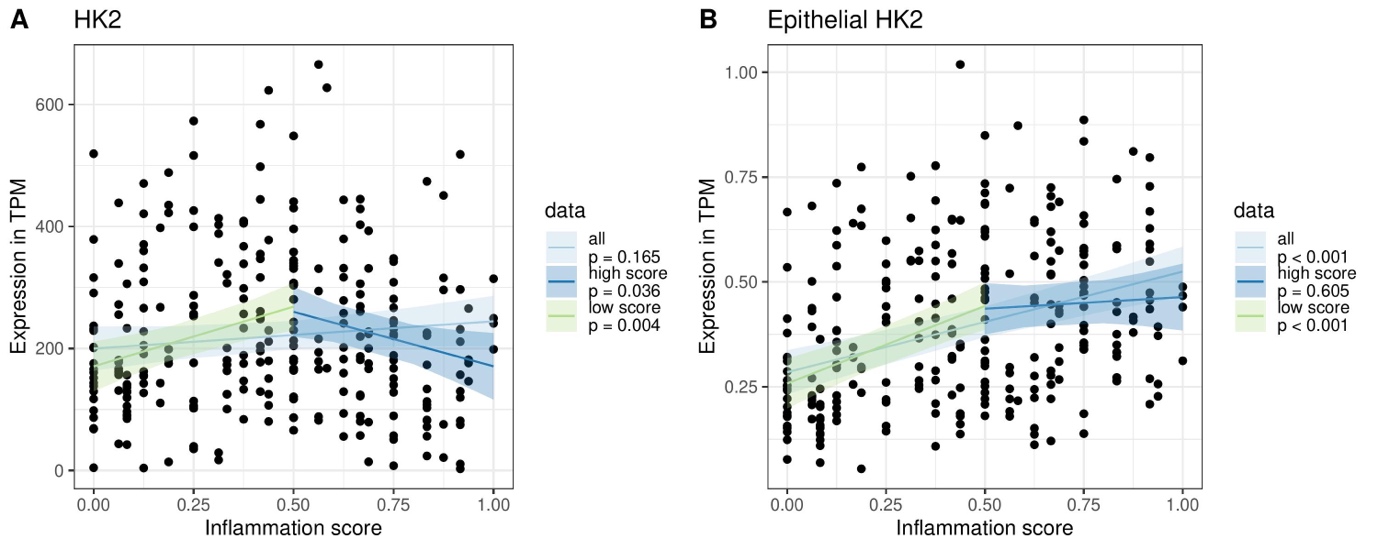


**Additional file 7: Figure S4: Correlation analysis for HK2 expression and inflammation score.** Three different linear mixed effect models (PatientID as random factor) were fitted to the **(A)** *HK2* and the **(B)** epithelial *HK2* expression. First, all points were used to estimate the overall effect of the data and inflammation score. Afterwards the data set was split into “low scores” (inflammation score < 0.5), "high scores” (inflammation score >0.5) and fitted models for these data subsets. While the first model over all data showed no significant association between *HK2* expression and inflammation, there is a positive association at “low scores” and a negative association at “high scores” (see also Additional file 8: Table S4) indicating that *HK2* expression increases with inflammation during lower disease scores and then decreases at higher disease scores. For epithelial *HK2* expression there is a positive association for all data and “low inflammation scores”, while the at “high inflammation scores” there is no significant association, but epithelial *HK2* expression remained elevated. This therefore might indicate a saturation of the epithelial *HK2* expression at high inflammation.
