# Supplementary material for: Hexokinase 2 expression in apical enterocytes correlates with inflammation severity in patients with inflammatory bowel disease
Source: BMC Med. 2024 Oct 23;22:490. doi: 10.1186/s12916-024-03710-7 (PMC11515617; doi:10.1186/s12916-024-03710-7)
Supplement: Supplementary file 8 — Additional file 8: Table S4. Comparison of linear mixed model statistics for the coefficients explaining HK2 and epithelial HK2 expression with inflammation scores for models created with all data, low (score <0.5) or high (score >0.05) inflammation scores only. [file 12916_2024_3710_MOESM8_ESM.docx]

**Additional file 8: Table S4: Comparison of linear mixed model statistics for the coefficients explaining HK2 and epithelial *HK2* expression with inflammation scores for models created with all data, low (score <0.5) or high (score >0.05) inflammation scores only.**

| **Object** | **Model** | **Estimate** | **Std. Error** | **DF** | **t-value** | **p-value** | **adjusted p-value** |
| --- | --- | --- | --- | --- | --- | --- | --- |
| **HK2** | **All data** | 44.73 | 32.07 | 179.94 | 1.395 | 0.165 | 0.165 |
|  | **Low score** | 195.86 | 59.48 | 154.44 | 3.293 | 0.0123 | 0. 003 |
|  | **High score** | -178.6 | 78 | 114.87 | -2.29 | 0.0239 | 0.0358 |
| **epithelial HK2** | **All data** | 0.24 | 0.043 | 235.70 | 5.546 | 7.8E-8 | 2.3E-7 |
|  | **Low score** | 0.37 | 0.077 | 165.99 | 4.775 | 3.9E-6 | 5.89E-6 |
|  | **High score** | 0.06 | 0.107 | 125.55 | 0.519 | 0.605 | 0.605 |
